# Recurrent *NUS1* canonical splice donor site mutation in two unrelated individuals with epilepsy, myoclonus, ataxia and scoliosis - a case report

**DOI:** 10.1186/s12883-019-1489-x

**Published:** 2019-10-27

**Authors:** Kouhei Den, Yosuke Kudo, Mitsuhiro Kato, Kosuke Watanabe, Hiroshi Doi, Fumiaki Tanaka, Hirokazu Oguni, Satoko Miyatake, Takeshi Mizuguchi, Atsushi Takata, Noriko Miyake, Satomi Mitsuhashi, Naomichi Matsumoto

**Affiliations:** 1 Department of Human Genetics, Yokohama City University Graduate School of Medicine, 3-9 Fukuura, Kanazawa, Yokohama, 236-0004 Japan; 2 Department of Neurology, Yokohama Brain and Spine Center, Yokohama, 235-0012 Japan; 3 Department of Pediatrics, Showa University School of Medicine, Tokyo, 142-8555 Japan; 4 Department of Neurology and Stroke Medicine, Yokohama City University Graduate School of Medicine, Yokohama, 236-0004 Japan; 5 Department of Pediatrics, Tokyo Women’s Medical University, Tokyo, 162-8666 Japan; 6 Clinical Genetics Department, Yokohama City University Hospital, Yokohama, 236-0004 Japan

**Keywords:** Whole-exome sequencing, *NUS1*, Epilepsy

## Abstract

**Background:**

We encountered two unrelated individuals suffering from neurological disorders, including epilepsy and scoliosis.

**Case presentation:**

Whole-exome sequencing identified the same recurrent, de novo, pathogenic variant in *NUS1* [NM_138459.4:c.691 + 1C > A] in both individuals. This variant is located in the conserved *cis*-prenyltransferase domain of the nuclear undecaprenyl pyrophosphate synthase 1 gene (*NUS1)*, which encodes the Nogo-B receptor, an essential catalyst for protein glycosylation. This variant was confirmed to create a new splice donor site, resulting in aberrant RNA splicing resulting in a 91-bp deletion in exon 3 in both individuals. The mutant mRNA was partially degraded by nonsense mediated mRNA decay. To date, only four de novo variants and one homozygous variant have been reported in *NUS1*, which cause developmental and epileptic encephalopathy, early onset Parkinson’s disease, and a congenital disorder of glycosylation. Seven patients, including our two patients, have presented with epileptic seizures and intellectual disabilities.

**Conclusions:**

Our study strongly supports the finding that this recurrent, de novo, variant in *NUS1* causes developmental and epileptic encephalopathy with involuntary movement, ataxia and scoliosis.

## Background

The *NUS1* (nuclear undecaprenyl pyrophosphate synthase 1) gene encodes the Nogo-B receptor (NgBR) [1, 2], which interacts with dehydrodolichyl diphosphate synthase complex subunit (DHDDS) and promotes *cis*-prenyltransferase (*cis*-PTase) activity. NgBR is an essential catalyst of the dolichol monophosphate (Dol-P) biosynthetic machinery in eukaryotic cells [3, 4]. The well-conserved C-terminus domain of *cis*-PTase in NgBR has intrinsic effects for protein structure stabilization, in association with N-glycans. To date, four de novo *NUS1* variants have been reported in association with developmental and epileptic encephalopathy (DEE) and early-onset Parkinson’s disease, and one homozygous *NUS1* variant has been associated with a congenital disorder of glycosylation. Interestingly, two de novo variants [5] and a pair of compound heterozygous variants [6] in *DHDDS* have been reported in five patients with DEE seizures or congenital glycosylation defects, suggesting that pathogenic variants in the NgBR–DHDDS pathway may cause neurological disorders. Here, we report two unrelated Japanese patients with a novel, recurrent, de novo *NUS1* variant, who presented with epileptic seizures with involuntary movement, ataxia, intellectual disability and scoliosis.

## Case presentation

The patient 1 was the second child born to non-consanguineous, healthy parents. Her elder brother had febrile seizures during childhood. She was born spontaneously, at full term, with no asphyxia. Her birth weight was 2826 g (− 0.44 SD). She gained head control at 4 months of age and sat without support at 7 months of age. She experienced febrile seizures at 9 months of age and generalized tonic-clonic convulsions without fever at 14 months of age, at which time valproic acid (VPA) was administered. Tremulous myoclonus of the extremities was also observed. She walked without support at 20 months of age, spoke a meaningful word at 10 months of age and two-word phrases at 24 months of age. Her developmental quotient was 77 at 2 years of age. Her seizures occurred once per year until the age of 6 years and 5 months; however, an increase in the VPA dosage lessened an episode of convulsive attack, after which her seizures disappeared. Her electroencephalograms (EEG) showed 3-Hz, diffuse, spike-and-slow-wave complexes with a 7-Hz slow wave background at 8 years of age (Additional file 1: Figure S1A), which became worsened at 15 years of age. However, the treatment with VPA and LEV significantly lessened 3-Hz diffuse spike-and-wave complexes and only 3-Hz high-amplitude slow wave bursts were infrequently recorded during sleep at 17 years of age (Additional file 1: Figure S1B). Her brain magnetic resonance imaging (MRI) results were normal at 6 years of age (Additional file 1: Figure S1C, S1D) and at 15 years of age (Additional file 1: Figure S1E, S1F). At the age of 17 years, her height was 157.4 cm (− 0.12 SD) and her weight was 41.9 kg (− 1.42 SD). She had no dysmorphic features except for scoliosis which needed a surgical correction at 15 years of age. She showed dysgraphia, due to tremulous myoclonus of the bilateral extremities (Additional file 4: Movie S1). She showed no behavioral disorders, such as autistic spectrum disorders or attention deficit/hyperkinetic disorder.


**Additional file 4: Movie S1.** Involuntary movements of Patient 1 at 17 years of age. Patient 1 shows frequent eye blinking, multifocal facial twitching, tremulous myoclonus of the upper extremities, mild dysmetria, and clumsiness of diadochokinesis.


In patient 1, trio (sequencing with parents) WES was performed, and 5 de novo variants were detected (Additional file 5: Table S1 and Additional file 6: Table S2 and Additional file 7: Supplemental method). Patient 1 had a splice site variant in *NUS1* [NM_138459.4:c.691 + 1C > A] on chromosome 6q22.1 (chr6: 118,015,344). This variant was absent from public databases (allele frequency was 0 in ExAC, gnomAD, ESP6500, HGVD, ToMMo and in-house 575 Japanese exome controls). Multiple *in-silico* evaluation scores for predicting the pathogenicity of DNA sequence alternations suggest that this variant is deleterious: Mutation Taster (http://www.mutationtaster.org/) returned a value of disease causing; CADD (https://cadd.gs.washington.edu/) returned a value of 25.9; and Fathmm (http://fathmm.biocompute.org.uk/) returned a value of deleterious. This splice variant is predicted [7] to create a new splice donor site, which could change the reading frame and introduce a premature termination codon (PTC).

The patient 2 was born normally to non-consanguineous, healthy parents. His birth weight was 3550 g. At the age of 6, it was noticed he had speech delay, clumsiness of the hands, and involuntary movements of the hands when he used chopsticks. At the age of 8 years, he was suspected to have a cerebellar atrophy, with seizures. Occasionally, jerky movements of the limbs also appeared. From 14 years of age, he gradually developed a gait abnormality. At the age of 37 years, he was admitted to hospital. On physical examination, he showed flat foot and limb ataxia. Clonazepam was remarkably effective for treating his gait disturbance. Laboratory examinations were all normal, including lactic acid, pyruvic acid, vitamins, the thyroid gland, ceruloplasmin, copper, lipoproteins, amino acid analysis and leukocyte lysosome enzyme activities (α-galactosidase, β-galactosidase, β-hexosaminidase and arylsulfatase). Genetic testing for dentatorubral-pallidoluysian atrophy was negative. At the age of 40 years, myoclonic jerks of the limbs developed, in addition to ataxia. He was diagnosed as progressive myoclonus epilepsy with an unknown cause. At the age of 42 years, scoliosis became apparent. Starting at the age of 48 years, he began to require assistance with walking. At the latest examination (59 years), he showed intellectual disability (equivalent to that of a 6-year-old), excessive blinking due to tenseness, and profound action myoclonus of the limbs, which could be referred to “hyperkinésie volitionnelle”. His eye pursuit was saccadic, and his speech was explosive. Tendon reflexes were slightly increased, and no sensory disturbances were observed. An EEG analyzing jerk-locked back averaging potentials suggested that the myoclonus emerged from the cortex. Examinations of MRI, nerve conduction studies, conventional EEGs, and laboratory examinations of the cerebrospinal fluid and blood were almost within normal ranges (Additional file 2: Figure S2A, S2B). Increasing the dosage of clonazepam up to 12 mg (0.5 mg × 24 tablets/day) did not alleviate neurological symptoms; however, the oral administration of 50 mg baclofen remarkably lessened myoclonus and slightly improved gait disturbance.

**Fig. 1:**
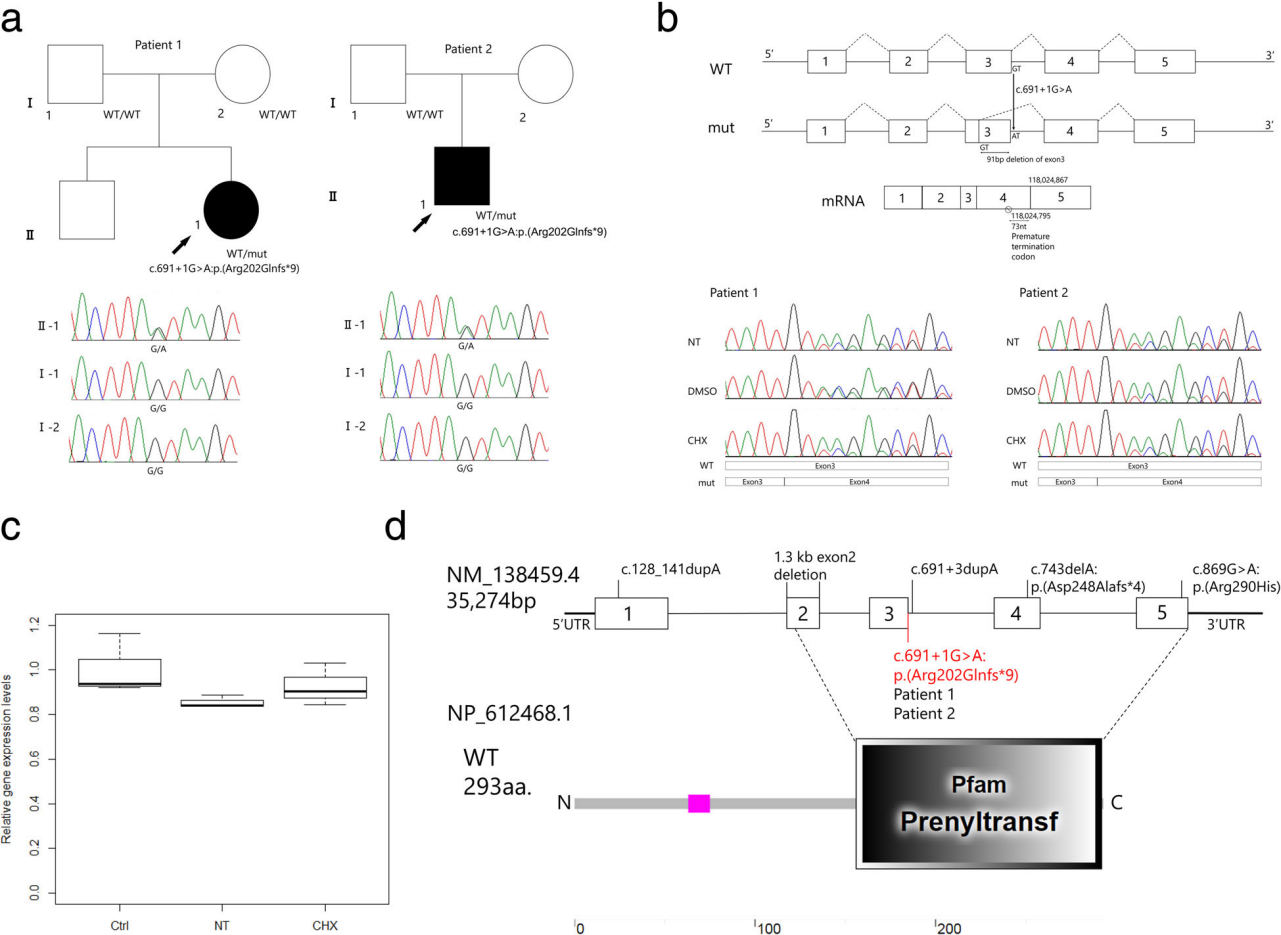
**a** Schematic presentation of the familial pedigrees of patients 1 and 2, with electropherograms of the heterozygous *NUS1* variant occurring de novo. **b** Schematic presentation of the *NUS1* gene structure, with identified variants that result in abnormal cDNA. Based on the cDNA sequencing, the variant [c.691 + 1G > A] creates a new splice donor site in the middle of exon 3 [c.601_602], resulting in the loss of a 91-bp section of the *NUS1* exon 3. **c** Comparison of the gene expression levels between a control and a patient. Patient LCLs were cultured either with no treatment (NT) or with cycloheximide (CHX) to test NMD involvement. Gene expression was normalized to that of GAPDH. Error bars represent the 10th to 90th percentiles. The vertical numbers (Y-axis) represent the levels of relative gene expression. **d** Pathogenic *NUS1* variants (including the current variant) mapped to the gene and the protein. A prenyltransferase domain is encoded by the middle of exons 2 to 5. Wild-type protein has 293 amino acids, and the prenyltransferase domain is composed of amino acids 156 to 292. This figure was designed using SMART software (http://smart.embl-heidelberg.de/)

We performed proband-only WES in patient 2 and detected a splicing site alteration variant in *NUS1* [c.691 + 1C > A] and a stop-gain variant in *SPTAN1* [c.2311G > T:p.(Glu771*)]. These two variants were confirmed by Sanger sequencing using the parents’ DNA, and only the *NUS1* variant [c.691 + 1G > A] occurred de novo (Fig. 1a and Additional file 7: Supplemental method). *SPTAN1* variant was inherited from healthy mother. Therefore, its pathogenicity should be minimal in the patient.

To test whether this variant causes aberrant splicing, we examined the cDNA from both individuals’ lymphoblastoid cell lines, which revealed that the mutant allele has a 1-base alternation in the splice donor site (chr6:118,015,344) creating a new splice donor site of GT in exon 3 (chr6:118,015,253), resulting in a 91-bp deletion in the *NUS1* exon 3 (Fig. 1b). Electropherograms of both individuals’ cDNA showed that a 91-bp region of exon 3 is missing. TA-cloning of the short RT-PCR product confirmed the same event in the mutant allele (Fig. 1b, Additional file 3: Figure S3A and Additional file 7: Supplemental method). This variant creates a new reading frame [c.601_691del:p.(Arg202Glnfs*9)] and produces a PTC at chr6:118,024,795 (73-bp upstream of the 3′ exon-exon junction) (Fig. 1b). According to the major rule of nonsense-mediated mRNA decay (NMD) [8], the mRNA of the mutant allele should be subjected to NMD. However, the results of both individuals’ cDNA sequencing chromatograms showed that cycloheximide (an NMD inhibitor) treatment did not recover the peak height of the electropherogram, suggesting that NMD is not involved. Thus, we performed a quantitative analysis using RT-PCR (see Additional file 7: Supplemental method). RT-PCR showed that the relative gene expression levels of *NUS1* was slightly reduced in the patients’ LCLs, with possible minor recovery following cycloheximide treatment (Fig. 1c). These results support the prediction that the transcribed mRNA of the mutant allele is only partially subjected to NMD.

## Discussion and conclusions

As the pLI score [9] of *NUS1* in the ExAC browser is 0.87, suggesting that an intolerance to *NUS1* loss-of-function variants, *NUS1* variants may lead to diseases through haploinsufficiency. Diseases-associated, autosomal-dominant loss-of-function variants in *NUS1* have been identified in patients with the following disorders: developmental delays and epileptic encephalopathy, [c.743delA:p.(Asp248Alafs*4)], [c.128_141dup:p.(Val48Profs*7)] and an approximately 1.3-kb deletion of exon 2 [5]; and a congenital disorder of glycosylation, [c.869G > A:p.(Arg290His)] [10]; and early-onset Parkinson’s disease, [c.691 + 3dupA] [11]. In addition, microdeletions involving *NUS1* have been linked to pediatric epilepsy [12, 13]. The four loss-of-function variants identified in *NUS1* occurred de novo (Table 1, Fig. 1d). These variants are located on the C-terminus *cis*-PTase domain, which is well-conserved in mammals. The *cis*-PTase domain interacts with DHDDS, and NgBR influences N-linked protein glycosylation via the regulation of *cis*-PTase activity [3] (Fig. 1d). A previous study [10] reported a homozygous variant, [c.869G > A:p.(Arg290His)] (Fig. 1d), in siblings with a congenital glycosylation disorder, congenital scoliosis and developmental delay, and refractory epilepsy. This mutation was found to decrease *cis*-PTase activity in the patients’ fibroblasts and caused a defect in the dolichol biosynthesis pathway. There are some phenotypic similarities among patients with *NUS1* variants, regardless of inheritance patterns. The two individuals with the de novo [c.691 + 1C > A] variant identified in our study share similar phenotypes: epilepsy, involuntary movement, ataxia, intellectual disability, and scoliosis (Table 1).

**Table 1:** Clinical features of individuals with *NUS1* variants

Origin	This study		Guo et al.	Hamdan et al.			Park et al.	
Individuals	Patient 1	Patient 2	Patient 3	Patient 4	Patient 5	Patient 6	Patient 7	Patient 8
Mutation	c.691 + 1C > A	c.691 + 1C > A	c.691 + 3dupA	c.743delA	c.128_141dup	exon 2 deletion, 1.3 kb	c.869G > A	c.869G > A
Amino acid change	c.601_691del:p.(Arg202Glnfs*9)	c.601_691del:p.(Arg202Glnfs*9)	–	(p.Asp248Alafs*4)	(p.Val48Profs*7)	–	p.Arg290His	p.Arg290His
Zygosity	de novo	de novo	de novo	de novo	de novo	de novo	homozygous	homozygous
Age	17 years	59 years	26 years	8 years 9 months	15 years	29 years	Deceased at 29 months	4 years
Sex	Female	Male	Female	Male	Male	Female	Male	Male
Consanguinity	No	No	*N/A*	*N/A*	No	No	*N/A*	*N/A*
Ethnicity	Japanese	Japanese	Chinese Han	*N/A*	French-Canadian	Caucasian	Czechs	Czechs
Birth weight	2826 g (−0.44 SD)	3500 g	*N/A*	*N/A*	2489 g	*N/A*	*N/A*	*N/A*
Birth length	N/A	56 cm	*N/A*	*N/A*	*N/A*	*N/A*	*N/A*	*N/A*
Age at seizure onset	9 months	8 years	16 years	12 months	10 months	2.5 years	11 months	7 months
Type of seizures	Febrile seizure at 9 months, generalized tonic-clonic convulsion without fever at 14 months, status epilepticus at 6 years 3 months	Loss of consciousness without convulsion at 8 years	*N/A*	Generalzed myoclonic epilepsy, convulsive epilepsy, nocturnal jerks	Myoclonic absences with behavioural arrest, facial and palpebral myoclonus	Myoclonic absences with behavioural arrest and eyelid flutters, as well as limb myoclonus	Tonic-clonic seizures, refractory epilepsy and recurrent attacks of “status epilepticus”	Refractory epilepsy, severe seizure
Frequency of seizures	Seizure-free since 6 years of age	N/A	*N/A*	*N/A*	5 times a day, lasting 5–10 s	1–2 times a week	N/A	N/A
Type of EEG	3-Hz, diffuse, spike-and-slow-wave, complexed with 7-Hz, slow wave background	8–9 Hz slow α rhythm background with no epileptiform activity	*N/A*	Bifrontal epileptiform activity	Diffuse background slowing, with rhythmic, bifrontal, high-amplitude theta discharges	Generalized spike-wave and poly-spike wave activity	N/A	N/A
Effective medicines for seizures	Valproic acid was effective for seizures, levetiracetam lessened 3-Hz, diffuse, spike-and-slow wave complexes	Myoclonus lessened with 50 mg baclofen	*N/A*	Levetiracetam	*N/A*	Relatively well-controlled with a combination of valproic acid, lamotrigine and clonazepam	N/A	N/A
Brain MRI	Normal at 20 months,slight cerebellaratrophy at 14 years	Normal	Normal	Normal (2 years 3 months)	Normal (8 years)	Normal	N/A	Severe cortical atrophy
Intellectual disability	Mild to moderate	Moderate	*N/A*	Moderate	Moderate	Mild	Yes	N/A
Language delay	Mild (speaking two-word sentences at 2 years)	Yes	No	Yes	Mild	No	N/A	N/A
Developmental delay	Mild psychomotor delay	No	*N/A*	Yes	Yes	Mild motor delay	Yes	N/A
Ataxia	Yes	Yes	*N/A*	Yes	No	No	N/A	N/A
Autsim	No	No	*N/A*	*N/A*	Yes	*N/A*	N/A	N/A
Scoliosis	Yes (operation at 15 years of age)	Yes	*N/A*	*N/A*	*N/A*	*N/A*	Yes, congenital	Yes, congenital
Hypotonia	No	No	*N/A*	*N/A*	*N/A*	*N/A*	Severe	Severe
Dysmorphic features	No	No	*N/A*	*N/A*	No	*N/A*	Microcephaly	Microcephaly
Additional features	Dysgraphia due to tremulous myoclonus of bilateral extremities	Eye pursuits were saccadic, hyperkinesie volitionelle-like movement, cortical myoclonus	Parkinson’s disease, asymmetric onset, bradykinesia, resting tremor in limbs, mild gait difficulties	–	–	Eye pursuits were saccadic, but saccades were normal	Histophathological examination of autopsy tissue revealed non-specific neuronal loss in brain cortex and cerebellum	–

*N/A* Not available, Not assessed

The newly identified variant in this study created a PTC 73 nucleotides upstream of the last exon-exon junction (Fig. 1b), which should be subjected to NMD according to the < 50-bp rule of escaping from NMD [8]. We speculate that NMD was not fully induced in the studied patients, based on the results of our comparative gene expression analyses (Fig. 1c). A previous study [11] of the splice site mutation [c.691 + 3dupA] showed a larger decrease in mRNA expression compared with the results in our study. This inconsistency may be due to differences in the variants or to different cells being tested. Although the mutant allele is expressed, our study predicted that the variant results in aberrant splicing, resulting in the expressed NgBR lacking an important functional domain.

In conclusion, we found a recurrent, de novo variant in *NUS1*, [c.691 + 1C > A], in two unrelated individuals. Both individuals had a similar phenotype: epil7epsy, involuntary movement, ataxia, intellectual disability, and scoliosis. This study strongly suggests that loss-of-function variants in *NUS1* that result in the loss of the *cis*-PTase domain in the C-terminus of NgBR may cause neurological disorders with scoliosis.

## Supplementary information

**Additional file 1: Figure S1.** (EEG and MRI of Patient 1). Interictal electroencephalogram (EEG) of Patient 1. A 3-s burst of 3-Hz, frontal-dominant, diffuse, spike-and-slow wave complexes is shown on an EEG at the age of 8 years (A). an EEG at 17 years (B) shows a similar burst of 3-Hz, high-amplitude (> 300 μV), slow waves, but no noticeable spike discharges. Brain MRIs for Patient 1 at the age of 6 years (C and D) and 15 years (E and F). T2-weighted axial images (C and E) and T1-weighted midsagittal images (D and F) show normal findings.

**Additional file 2: Figure S2.** (MRI of Patient 2). Brain MRIs of Patient 2 at the age of 56 years (A and B). T2-weighted axial image (A) and T1- weighted midsagittal image (B) show normal findings.

**Additional file 3: Figure S3.** A Agarose gel electrophoresis of cDNA fragments. B Gene expression levels of *NUS1*, normalized to those of actin. RT-PCR primers were same as those reported by Guo et al. [11]. Gene expression level was normalized to the expression level of actin: 5′-CCGGAAGATGGAAAAGCAGA-3′ (forward), 5′-TCCTTTCCTCCACAAGCCT-3′ (reverse). Gene expression levels were compared to those of control (ctrl) and no cycloheximide treatment (NT) conditions.

**Additional file 5: Table S1.** Process of variant filtering for patient 1.

**Additional file 6: Table S2.** Detailed information for the five de novo variants found in patient 1, including in silico prediction scores and allele frequencies.

**Additional file 7:** Supplemental Method.

## Data Availability

The datasets generated and/or analyzed during the current study are not publicly available but are available from the corresponding author on reasonable request.

## Abbreviations

*cis*-PTase: *cis*-prenyltransferase
DEE: Developmental and epileptic encephalopathy
DMSO: Dimethyl sulfoxide
EEG: Electroencephalogram
LCL: Lymphoblastoid cell lines
MRI: Magnetic resonance imaging
NgBR: Nogo-B receptor
NMD: Nonsense mediated mRNA decay
NUS1: Nuclear undecaprenyl pyrophosphate synthase 1
PTC: Premature termination codon
SNV: Single nucleotide variant
VPA: Valproic acid
WES: Whole-exome sequencing
