# Supplementary figures and images for: Recurrent NUS1 canonical splice donor site mutation in two unrelated individuals with epilepsy, myoclonus, ataxia and scoliosis - a case report
Source: BMC Neurol. 2019 Oct 27;19:253. doi: 10.1186/s12883-019-1489-x (PMC6815447; doi:10.1186/s12883-019-1489-x)

8y

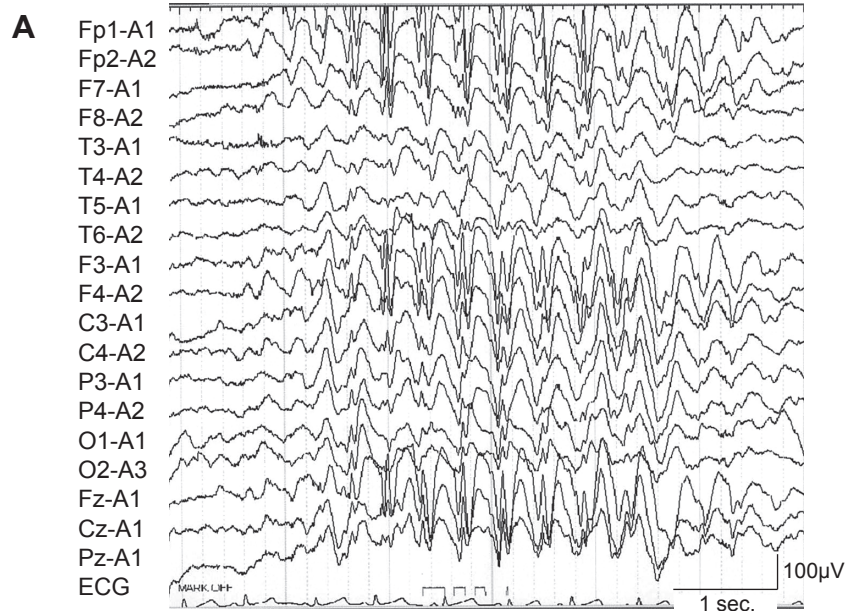

17y

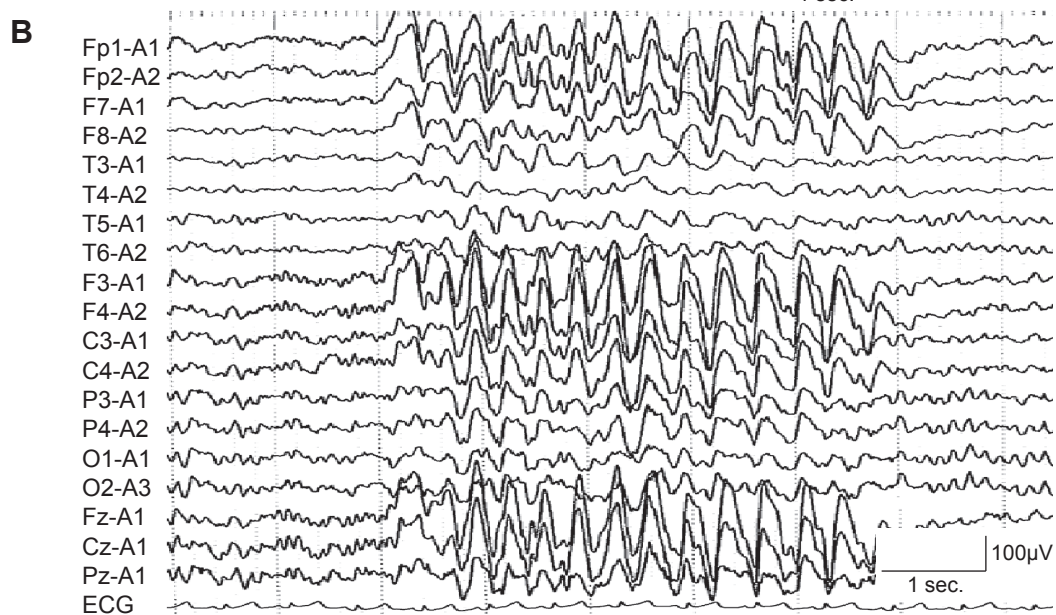

6y

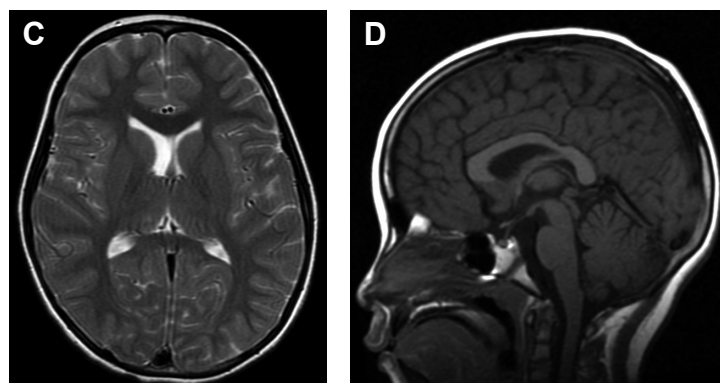

15y

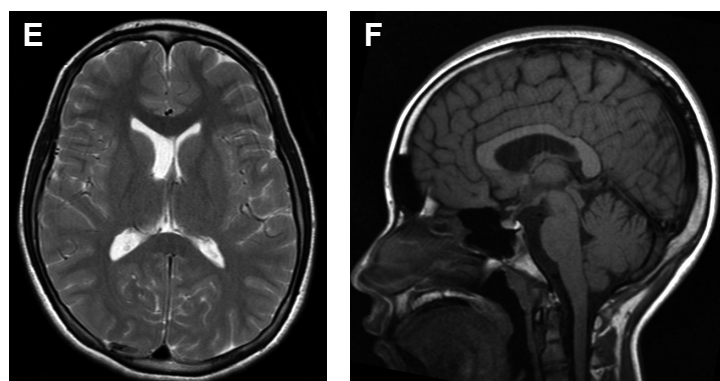

Supplement: Supplementary file 2 — Additional file 1: Figure S1. (EEG and MRI of Patient 1). Interictal electroencephalogram (EEG) of Patient 1. A 3-s burst of 3-Hz, frontal-dominant, diffuse, spike-and-slow wave complexes is shown on an EEG at the age of 8 years (A). an EEG at 17 years (B) shows a similar burst of 3-Hz, high-amplitude (> 300 μV), slow waves, but no noticeable spike discharges. Brain MRIs for Patient 1 at the age of 6 years (C and D) and 15 years (E and F). T2-weighted axial images (C and E) and T1-weighted midsagittal images (D and F) show normal findings. [file 12883_2019_1489_MOESM1_ESM.pdf]

A

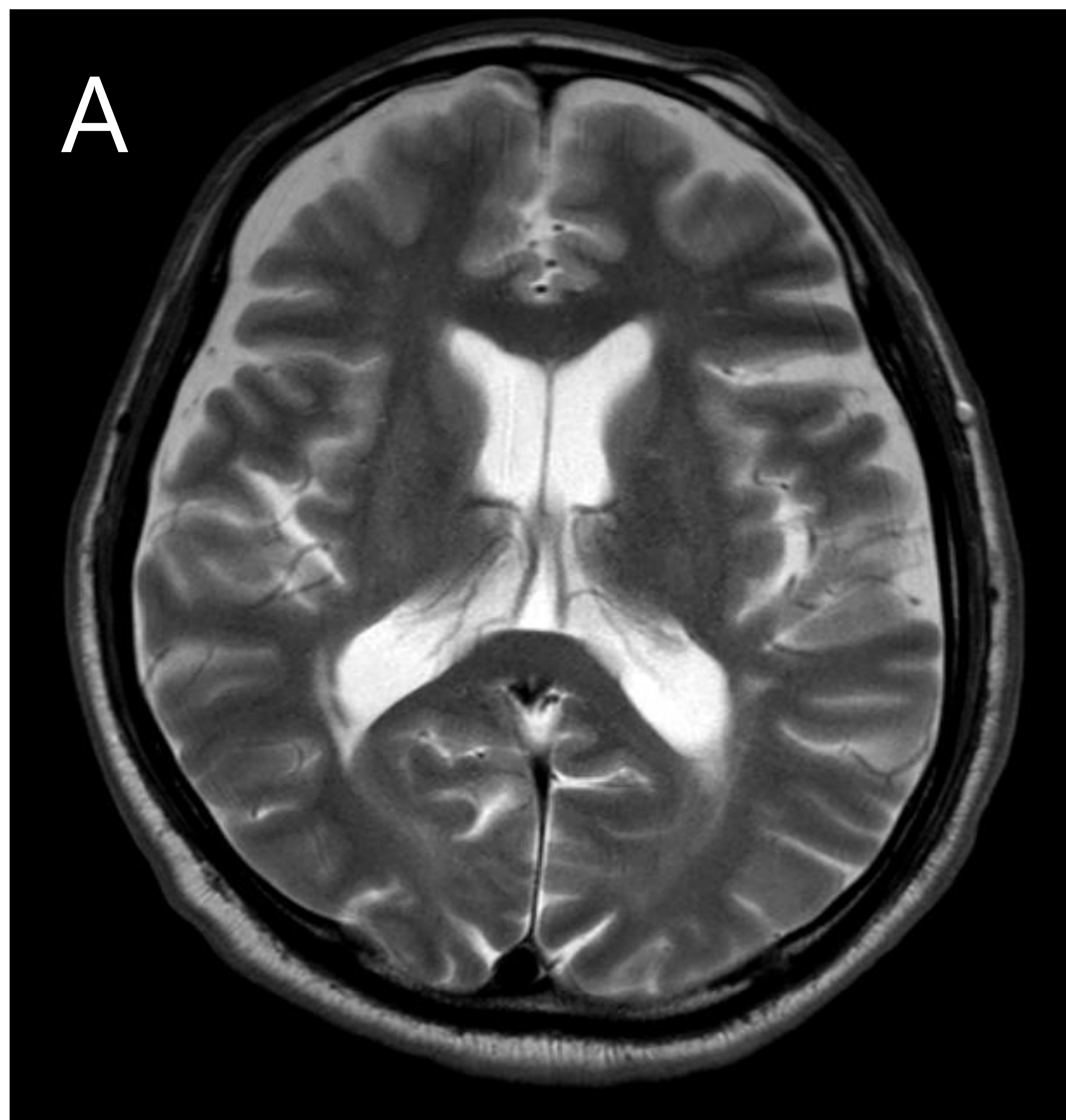

B

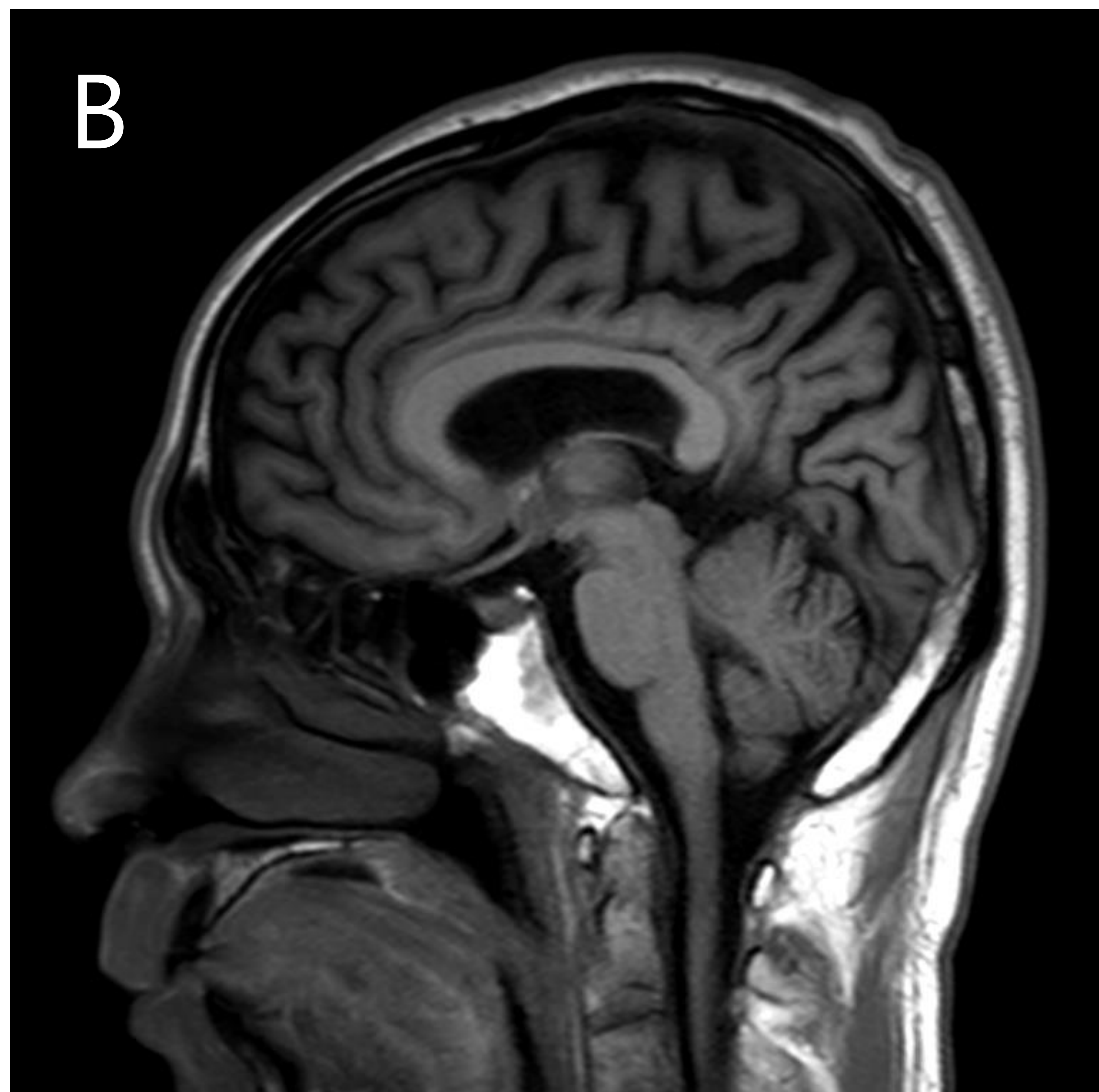

56y

Supplement: Supplementary file 3 — Additional file 2: Figure S2. (MRI of Patient 2). Brain MRIs of Patient 2 at the age of 56 years (A and B). T2-weighted axial image (A) and T1- weighted midsagittal image (B) show normal findings. [file 12883_2019_1489_MOESM2_ESM.pdf]
