# Supplementary material for: Recurrent NUS1 canonical splice donor site mutation in two unrelated individuals with epilepsy, myoclonus, ataxia and scoliosis - a case report
Source: BMC Neurol. 2019 Oct 27;19:253. doi: 10.1186/s12883-019-1489-x (PMC6815447; doi:10.1186/s12883-019-1489-x)

A

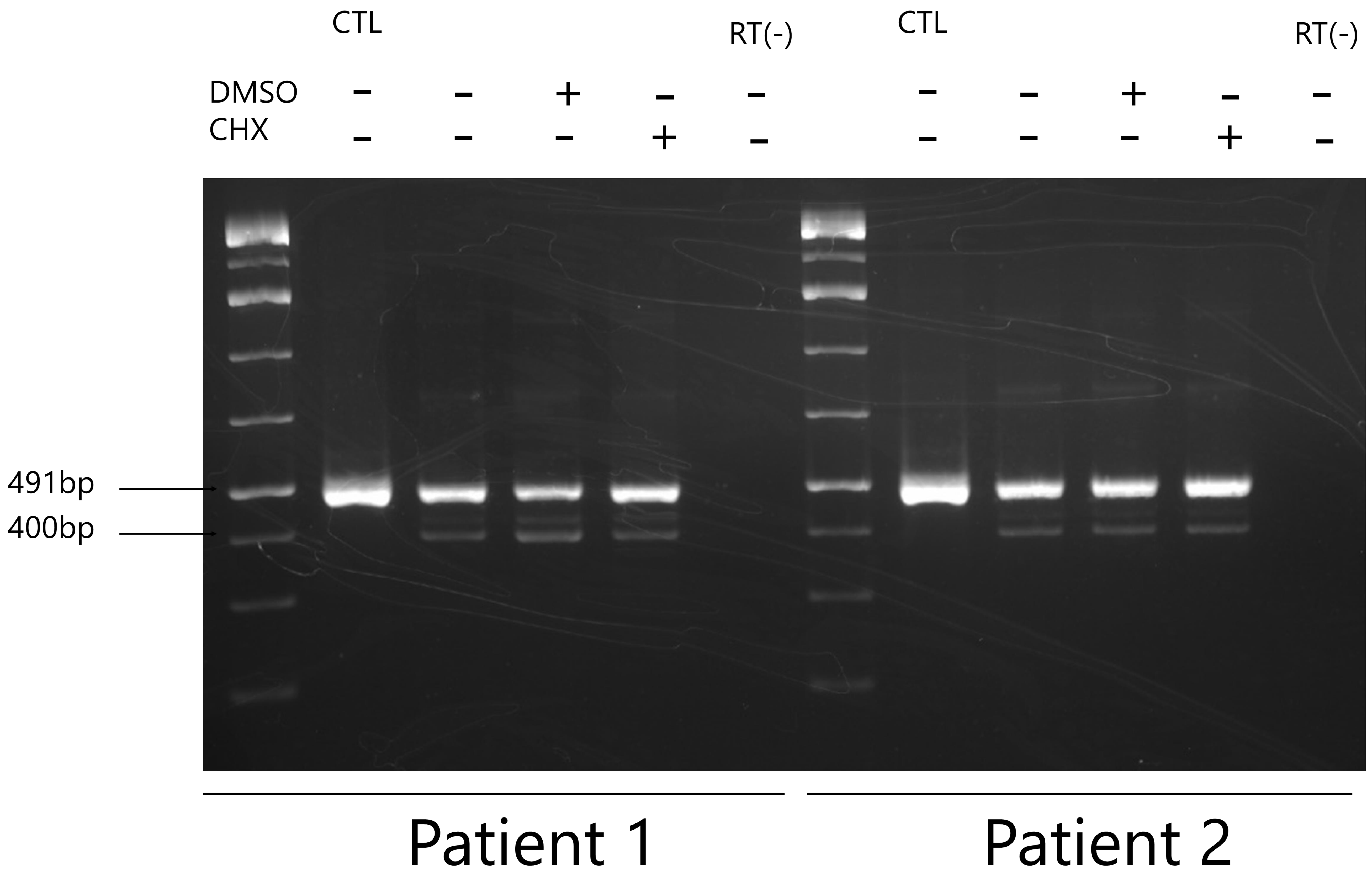

DMSO:Dimethyl sulfoxide
CHX:Cycloheximide
CTL:control
RT(-):No reverse transcription reaction

B

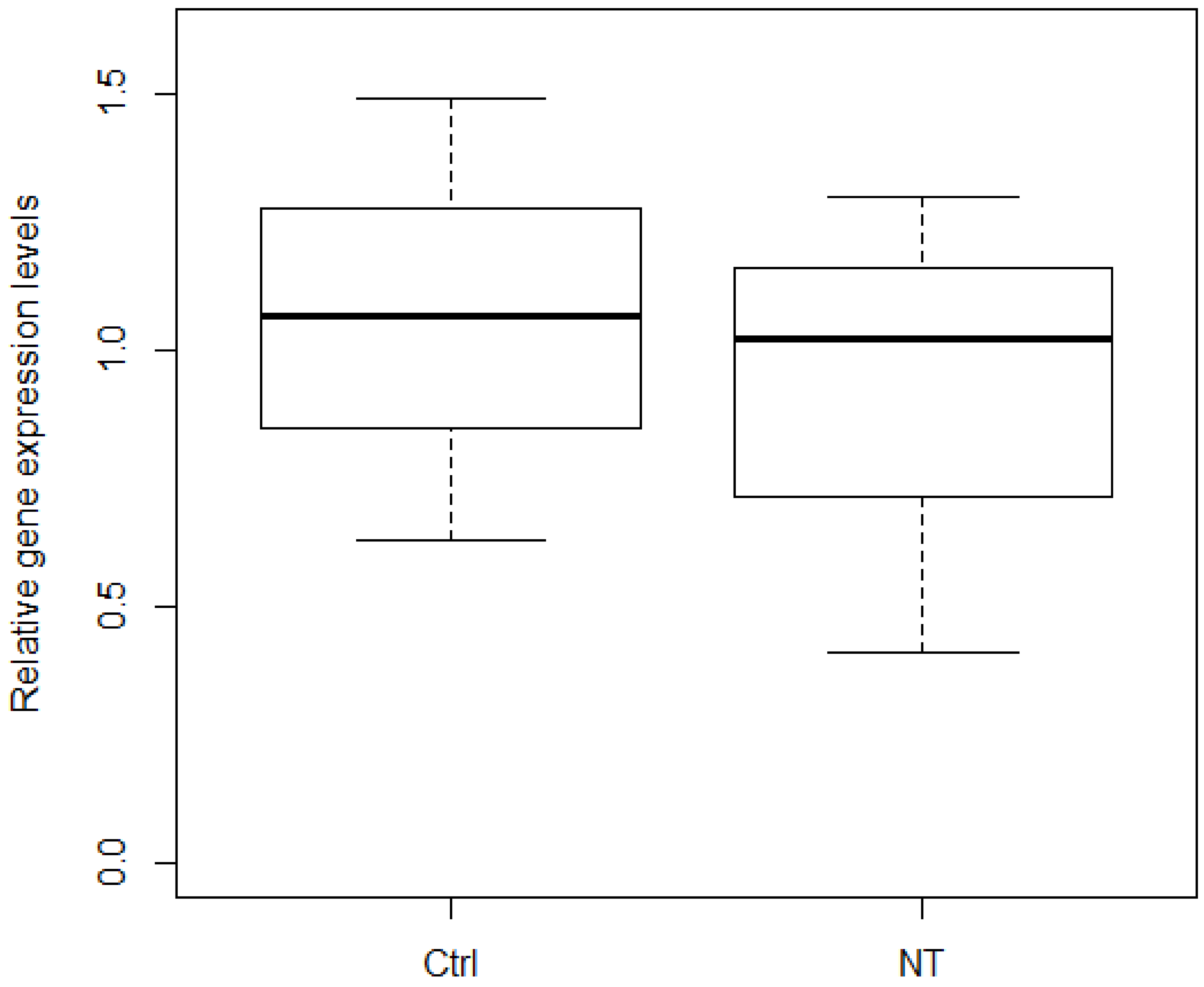

Supplement: Supplementary file 4 — Additional file 3: Figure S3. A Agarose gel electrophoresis of cDNA fragments. B Gene expression levels of NUS1, normalized to those of actin. RT-PCR primers were same as those reported by Guo et al. [11]. Gene expression level was normalized to the expression level of actin: 5′-CCGGAAGATGGAAAAGCAGA-3′ (forward), 5′-TCCTTTCCTCCACAAGCCT-3′ (reverse). Gene expression levels were compared to those of control (ctrl) and no cycloheximide treatment (NT) conditions. [file 12883_2019_1489_MOESM3_ESM.pdf]
