# Supplementary material for: Recurrent NUS1 canonical splice donor site mutation in two unrelated individuals with epilepsy, myoclonus, ataxia and scoliosis - a case report
Source: BMC Neurol. 2019 Oct 27;19:253. doi: 10.1186/s12883-019-1489-x (PMC6815447; doi:10.1186/s12883-019-1489-x)
Supplement: Supplementary file 7 — Additional file 7: Supplemental Method. [file 12883_2019_1489_MOESM7_ESM.docx]

***Supplemental Method***

**Whole-exome sequencing (WES)**

Participants’ genomic DNA was extracted from peripheral blood leukocytes. DNA concentrations (1 µg genomic DNA) were measured with a NanoDrop ND-1000 (Thermo Fisher Scientific, Wilmington, DE, USA) and a Qubit 3.0 Fluorometer (Invitrogen, Carlsbad, CA, USA). WES was performed as previously described ([1](#_ENREF_7)). Briefly, DNA were sheared, and exonic and flanking regions were captured with a SureSelectXT Human All Exon v6 Kit (Agilent Technologies, Santa Clara, CA, USA). Prepared libraries were sequenced with a Hiseq2500 sequencing system (Illumina, San Diego, CA, USA). Raw reads were aligned with a human GRCh37/hg19 reference using NovoAlign ver.3.02.13 (http://www.novocraft.com/). SAM files were converted to BAM files by SAMtools (https://github.com/samtools/samtools). PCR duplications were removed by Picard (http://broadinstitute.github.io/picard/). Realignments around indel and the recalibration of the base quality score were performed with GATK ver.3.7 (http://broadinstitute.org/gatk/). Variant annotations were performed with ANNOVAR (http://annovar.openbioinformatics.org). Allele frequencies of called variants were assessed using the following public databases; NHLBI GO Exome Sequencing Project (ESP6500; http://evs.gs.washington.edu/EVS/), Exome Aggregation Consortium (ExAC; http://exac.broadinstitute.org/), the Human Genetic Variation Database (HGVD; http://www.hgvd.genome.med.kyoto-u.ac.jp/), Tohoku Medical Megabank Organization (ToMMo; http://www.megabank.tohoku.ac.jp/), and in-house 575 Japanese exome controls.

**Variant filtering**

We were able to obtain more than 20 reads in >95% of the targeted region. To isolate rare variants in patient 1, we excluded minor allele frequencies >1% in the public databases and the in-house 575 Japanese exome controls for the first-round variant screening. In addition, synonymous ingle nucleotide variants (SNVs) were excluded, and ±3-bp borders of splice sites were kept.

**Sanger sequencing**

The PCR primer pair for the *NUS1* variant [NM_138459.4:c.691+1G>A] was as follows: 5’-TGGCAGTGAAGGTGCTGTCT-3’ (forward), and 5’-CATGCGAGTGAGTGGCTACA-3’ (reverse), for 352-bp fragments. Ex Taq DNA Polymerase (TaKaRa, Shiga, Japan) was used, with the following PCR conditions: 1 cycle at 94°C for 30 sec, 29 cycles at 98°C for 10 sec, 60°C for 30 sec, and 72°C for 30 sec, 1 cycle at 72°C for 1 min, and holding at 15°C. The purified PCR fragments were sequenced with the BigDye Terminator v3.1 Cycle Sequencing kit (Applied Biosystems, Foster City, CA, USA), using the 3130xl genetic analyzer. Microsatellite haplotype analysis using different 12 fluorescent markers was applied to patient 2 using GeneMapper Software 5 (Applied Biosystems).

**Reverse-transcription PCR and quantitative real-time PCR**

Lymphoblastoid cell lines (LCLs) from the two patients’ lymphocytes were extracted and cultured for 4 hours under three conditions: no treatment, 0.0003% dimethyl sulfoxide (DMSO), and cycloheximide dissolved in DMSO (30 µg/ml). RNA was extracted by an RNeasy Plus Mini Kit (QIAGEN, Hilden, Germany), and total RNA was subjected to reverse-transcription PCR using SuperScript III (Invitrogen) for first-strand cDNA synthesis. Primers were designed for 491-bp fragments, as follows: 5’-AGCGTCTACGACCACCAAGG-3’ (forward) and 5’-GACCACTACTTTCCCAGACGC-3’ (reverse). Amplified cDNA fragments were sequenced using the 3130xl genetic analyzer with the same primer pair.

For patient 1, quantitative real-time PCR was performed using a Rotor-Gene SYBR Green PCR Kit and Rotor-Gene Q (QIAGEN). PCR was performed using 35 cycles at 95°C for 5 min, 95°C for 10 sec, and 60°C for 30 sec. The primer pair used for *NUS1* was the same pair reported by Guo *et al.* ([2](#_ENREF_8)), as follows: 5’- AGCCTCGTGGTGTGGTGTAT- 3’ (forward) and 5’-GCCCAGAAGTTCTTGCTGTT-3’ (reverse). Gene expression level was normalized to that of glyceraldehyde-3-phosphate dehydrogenase (*GAPDH*), which used the following primary pair: 5’-GCCAAAAGGGTCATCATCTC-3’ (forward), and 5’-TTCACACCCATGACGAACAT-3’ (reverse). The comparative delta C(T) method ([3](#_ENREF_9)) was used to analyze quantitative gene expression.

**TA Cloning**

Amplified cDNA fragments were separated by 1% agarose-gel electrophoresis in 1× Tris/Borate/EDTA (TBE) buffer, at 135 V for 90 min. DNA bands were cut out of the agarose-gel and purified using EconoSpin All-in-One Mini Spin Columns (Epoch Life Science, Missouri City, TX, USA). DNA fragments were inserted into the plasmid using the TOPO TA Cloning Kit (Invitrogen) and sequenced with the M13 forward primer: 5’-GTAAAACGACGGCCAG-3’ (Invitrogen).

**References**

1. Hamanaka K, Miyatake S, Zerem A, Lev D, Blumkin L, Yokochi K, et al. Expanding the phenotype of IBA57 mutations: related leukodystrophy can remain asymptomatic. Journal of human genetics. 2018;63(12).
2. Guo JF, Zhang L, Li K, Mei JP, Xue J, Chen J, et al. Coding mutations in NUS1 contribute to Parkinson's disease. Proceedings of the National Academy of Sciences of the United States of America. 2018;115(45).
3. Schmittgen TD, Livak KJ. Analyzing real-time PCR data by the comparative C(T) method. Nature protocols. 2008;3(6).
